# Field-deployable multiplex detection method of SARS-CoV-2 and influenza virus using loop-mediated isothermal amplification and DNA chromatography

**DOI:** 10.1371/journal.pone.0285861

**Published:** 2023-05-16

**Authors:** Kyoko Hayashida, Alejandro Garcia, Lavel Chinyama Moonga, Tatsuki Sugi, Kodera Takuya, Mitsuo Kawase, Fumihiro Kodama, Atsushi Nagasaka, Nobuhisa Ishiguro, Ayato Takada, Masahiro Kajihara, Naganori Nao, Masashi Shingai, Hiroshi Kida, Yasuhiko Suzuki, William W. Hall, Hirofumi Sawa, Junya Yamagishi

**Affiliations:** 1 International Institute for Zoonosis Control, Hokkaido University, Sapporo, Japan; 2 International Collaboration Unit, International Institute for Zoonosis Control, Hokkaido University, Sapporo, Japan; 3 Department of Paraclinical Studies, School of Veterinary Medicine, University of Zambia, Lusaka, Zambia; 4 UCD Centre for Experimental Pathogen Host Research, University College Dublin, Belfield, Ireland; 5 TBA Co., LTD, Sendai, Japan; 6 Sapporo City General Hospital, Sapporo, Japan; 7 Division of Infection Control, Hokkaido University Hospital, Sapporo, Japan; 8 One Health Research Center, Hokkaido University, Sapporo, Japan; 9 Institute for Vaccine Research and Development, Hokkaido University, Sapporo, Japan; 10 Centre for Research in Infectious Diseases, School of Medicine and Medical Science, University College Dublin, Belfield, Ireland; 11 Global Virus Network, Baltimore, Maryland, United States of America; Huadong Research Institute for Medicine and Biotechniques, CHINA

## Abstract

A novel multiplex loop-mediated isothermal amplification (LAMP) method combined with DNA chromatography was developed for the simultaneous detection of three important respiratory disease-causing viruses: severe acute respiratory syndrome coronavirus 2 (SARS-CoV-2), influenza A virus, and influenza B virus. Amplification was performed at a constant temperature, and a positive result was confirmed by a visible colored band. An in-house drying protocol with trehalose was used to prepare the dried format multiplex LAMP test. Using this dried multiplex LAMP test, the analytical sensitivity was determined to be 100 copies for each viral target and 100–1000 copies for the simultaneous detection of mixed targets. The multiplex LAMP system was validated using clinical COVID-19 specimens and compared with the real-time qRT-PCR method as a reference test. The determined sensitivity of the multiplex LAMP system for SARS-CoV-2 was 71% (95% CI: 0.62–0.79) for cycle threshold (Ct) ≤ 35 samples and 61% (95% CI: 0.53–0.69) for Ct ≤40 samples. The specificity was 99% (95%CI: 0.92–1.00) for Ct ≤35 samples and 100% (95%CI: 0.92–1.00) for the Ct ≤40 samples. The developed simple, rapid, low-cost, and laboratory-free multiplex LAMP system for the two major important respiratory viral diseases, COVID-19 and influenza, is a promising field-deployable diagnosis tool for the possible future ‘twindemic, ‘ especially in resource-limited settings.

## Introduction

Since the emergence and rapid global spread of severe acute respiratory syndrome coronavirus 2 (SARS-CoV-2) in early 2020, coronavirus disease 2019 (COVID-19) continues to pose a serious threat to public health and negatively affect the global economy. Early diagnosis is critical for appropriate patient care and the prevention of disease spread. In most reference laboratories, the diagnosis of COVID-19 is confirmed by detecting SARS-CoV-2 RNA using real-time reverse transcription-PCR (qRT-PCR), which is considered the gold-standard method due to its high sensitivity and specificity. However, obtaining results often requires a few hours and well-equipped laboratories. Moreover, the costs of the equipment and reagents are high. As such, rapid diagnostic tests (RDTs) are frequently used, especially when a rapid result is required, such as in arrival testing at airports, or areas with limited laboratory access. The disadvantages of RDTs are lower sensitivity and specificity compared to qRT-PCR method, although variable performance has been reported [1].

Several novel diagnostic approaches have been developed for the detection of SARS-CoV-2 nucleic acids [2]. Among these, reverse transcription loop-mediated isothermal amplification (RT-LAMP) is a promising alternative rapid diagnostic tool with high sensitivity, specificity, speed, and simplicity [3]. Reverse transcription and amplification can be performed at a constant temperature for less than one hour. Regarding detection, LAMP amplification can be judged by changes in turbidity, fluorescence, or color by the naked eye. The simplicity of LAMP allows point-of-care testing, resulting in its wide application for the diagnosis of infectious diseases [4]. RT-LAMP methods for detecting SARS-CoV-2 are now commercially available, demonstrating moderate to excellent agreement with the gold-standard qRT-PCR [5, 6]. However, conventional RT-LAMP tests generally only detect a single pathogen; therefore, multiple reactions are required for the concurrent detection of more than two target pathogens. A multiplex LAMP assay that detects SARS-CoV-2 and influenza virus simultaneously has been reported [6], which utilized the Detection of Amplification by Releasing of Quenching (DARQ) method. DARQ uses a 5’-quencher-added FIP annealed to the 3’- fluorophore Fd, which is complementary to FIP. Following LAMP amplification, the primers detach and release Fd, which emits the fluorescence [7]. Although the system is robust and has great potential for the simultaneous detection of multiple pathogens, it still requires a fluorescence detector to obtain results, making it impractical for resource-limited laboratories.

DNA chromatography in a lateral flow (dipstick) format is originally established for the post-multiplex PCR visualization tool [8]. Recently, one of the simple formats of DNA chromatography “C-PAS,” developed by the Japanese company TBA, was applied for the visualization after multiplex LAMP amplification of *Sarcosystis* spp. and Shiga toxin-producing *Escherichia coli* from game meats [9], and *Plasmodium* spp. and *Rickettsia* spp. from human blood [10]. In this study, we developed a multiplex-LAMP assay combined with DNA chromatography to detect SARS-CoV-2, influenza A virus (IAV), and influenza B virus (IBV) simultaneously as a tool for rapid on-site diagnosis. We designed novel primer sets for detecting SARS-CoV-2 and optimized a multiplexing condition with the reported LAMP primer sets for detecting the influenza virus. This generic multiplex-LAMP assay is easy to perform, does not require expensive equipment, and is rapid. Thus, it has great potential as a point-of-care test for the simultaneous detection of COVID-19 and influenza.

## Materials and methods

### Viral RNA and *in vitro* transcribed RNA template

SARS-CoV-2 RNA was extracted from viral isolate (JPN/TY/WK-521 strain, Pango lineage: A, GISAID ID: EPI_ISL_408667) using Direct-zol RNA kits (Zymo research, Orange, CA). The virus was kindly provided by the National Institute of Infectious Diseases in Tokyo, Japan, and was propagated as previously described [11]. Influenza virus RNA was extracted from influenza A virus strains, A/Hokkaido/32/2011 (H1N1), and an influenza B virus strain, B/Hokkaido/2/2016 (Victoria lineage), respectively. To obtain the RNA of LAMP target regions, *in vitro* transcription RNA templates for SARS-CoV-2, IAV, and IBV were prepared by using *in vitro* transcription T7 kit (Takara, Shiga, Japan). The target regions were amplified with LAMP F3 and B3 primer for each virus (Table1) using QIAGEN OneStep RT-PCR Kit (Qiagen, Valencia, CA). The amplicons were cloned into a pGEMT-easy vector (Promega, Maddison, WI). *In vitro* transcribed RNA was treated by DNase, and purified by a Nucleospin RNA clean-up kit (Takara). The RNA concentration was measured by Qubit RNA HS kit and Qubit fluorometer (ThermoFisher, Waltham, MA), and copy numbers were calculated.

**Table 1:** Primer sets used for the multiplex LAMP.

Name of Primer	Primer Sequences (5’to 3’)	Ref position*
SARS-CoV-2_N_F3	TGGCTACTACCGAAGAGCT	28525–28543
SARS-CoV-2_N_B3	TGCAGCATTGTTAGCAGGAT	28722–28741
SARS-CoV-2_N_FIP	[F4 sequence tag] -C3-TCTGGCCCAGTTCCTAGGTAGTGAGAATTCGTGGTGGTGA	F1:28605–28626 F2:28548–28566
SARS-CoV-2_N_BIP	[biotin tag]-AGACGGCATCATATGGGTTGCA-GCGGGTGCCAATGTGATC	B1:28654–28675 B2:28703–28720
SARS-CoV-2_N_LF	TGGACTGGATCTTTCATTTTACCG	28567–28591
SARS-CoV-2_N_LB	ACTGAGGGAGCCTTGAATACACC	28676–28698
IAV_F3-1 IAV_F3-2	GACTTGAAGATGTCTTTGC GACTGGAAAGTGTCTTTGC	[13, 14]
IAV-B3-1 IAV-B3-2	TRTTATTTGGGTCTCCATT TRTTGTTTGGGTCCCCATT	
IAV-FIP	[F1 sequence tag]-C3-TTAGTCAGAGGTGACARRATTGCAGATCTTGAGGCTCTC	
IAV-BIP	[biotin tag]-TTGTKTTCACGCTCACCGTGTTTGGACAAAGCGTCTACG	
IAV-LF	GTCTTGTCTTTAGCCA	
IAV-LB	CMAGTGAGCGAGGACTG	
IBV-F3	GCAACCAATGCCACCATA	[13]
IBV-B3	TTCTCTCTTCAAGRGACATC	
IBV-FIP	[F3 sequence tag]-C3-TAGTCAAGGGCYCTTTGCCACTTTGAAGCAGGAATTCTGGA	
IBV-BIP	[biotin]-CAAGACCGCCTAAACAGACTAAACTTTTACTTTCAGGCTCACTT	
IBV-LF	TGAAAGYCTTTCATAGCAC	
IBV-LB	CAAGAATAAAGACTCACAAC	

Tag sequences F1, F3, F4 (obtained from TBA Co., LTD, Japan) and spacer C3 were added to the 5’ end of the primer set in FIP primers.

*Reference genome of SARS-CoV-2: hCoV-19/Wuhan/WIV04/2019 (WIV04)

### COVID-19 clinical specimens and influenza virus specimens

RNA was extracted from nasopharyngeal swabs which were collected in St. Vincent’s university hospital and Master Misericordiae University Hospital, Dublin, Ireland, in 2019 using the QIAamp Viral RNA Mini Kit (Qiagen) and used for the COVID-19 test. All specimens were originally collected after written informed consent had been obtained for the infectious diseases cohort project. In this study, we did not obtain any patient-related data, and only converted IDs were used. The use of the RNA sample secondarily for the validation of our study was approved by the St. Vincent’s University Hospital Ethics & Medical Research Committee and Master Misericordiae University Hospital (1/378/1397), and the Institutional Research Committee of Hokkaido University (jinjuu 2020–11).

A nasopharyngeal swab specimen was obtained from a healthy individual and was used to make pseudo-specimen spiked with influenza viruses. The RNA extracted from the original swab sample was all negative for IAV, IBV, and SARS-CoV-2 by qRT-PCR as described below. The viral titers were determined as plaque-forming units (PFU) using MDCK cells. A 140 μL of the nasal swab sample was pulsed with the known titers of IAV and IBV, and the 40 μL of RNA was extracted using QIAamp Viral RNA Mini kit (Qiagen).

### Primer for the reverse transcription-LAMP assay for SARS-CoV-2 and influenza viruses

Primers for detecting SARS-CoV-2 were designed using Primer Explorer V5 software (Eiken Chemical, Tokyo, Japan) targeting the nucleocapsid region of SARS-CoV-2. Out of the ten candidate primer sets of FIP/BIP/F3/B3, the best primer set was chosen which showed the fastest amplification time using a serially diluted SARS-CoV-2 DNA template. For the best candidate primer sets (S1 Table: primer candidate 1), LF and LB primers were further designed using Primer3 [12]. For the initial primer screening, the DNA template of the genome position at 28276–28975 (reference strain 2019-nCoV WHU01) was synthesized (Eurofins Genomics, Tokyo, Japan), and used as the LAMP reaction template. For influenza virus detection, we utilized already established primer sets [13, 14] with a modification in that we used primer sets without a quenching primer (QP). The primers were purchased from Thermo Fisher scientific unless specified.

### Reverse transcription-LAMP assay targeting a single target

Reverse transcription-LAMP assay as a single liquid format was performed as described [15]. Each 25 μl RT-LAMP reaction mix consisted of 3.75 U WarmStart RTx reverse transcriptase (New England Biolabs Inc., Ipswich MA), 4 U RNase inhibitor (Takara), 8 U Bst 2.0 WarmStart DNA polymerase (New England Biolabs Inc.), deoxyribonucleotide triphosphate at 1.4 mM each (dNTPs; Nippon Gene, Tokyo, Japan), 20 mM Tris-HCl pH10.0, 50 mM KCl, 6 mM MgSO4, 10 mM (NH_4_)_2_SO_4_, 160 mM Trehalose (Fujifilm Wako, Osaka, Japan) LAMP primer sets (FIP and BIP; 1.6 μM, F3 and B3; 0.2 μM, LF and LB; 0.8 μM), 120 μM colori-fluorometric indicator (CFI; 3 mM phydroxyl-naphtol blue; MP Biomedicals, Autora, OH, and 0.35% v/v GelGreen; 10,000x solution in DMSO, Biotium, Hayward, CA), 160 mM Trehalose (FUJIFILM Wako Pure Chemical, Osaka, Japan), 0.1% Triton X-100 containing DDW, and 2 μl of template RNA. FIP contained the sequence tag (F1, F3, F4) at the 5’ end, followed by the C3 spacer oligonucleotide modification before the F3 sequences for the DNA chromatography (Tohoku Bio Array: TBA, Sendai, Japan) hybridization. B3 primers had biotin modification at 5’ ends for the visualization on DNA chromatography by streptavidin beads. LAMP primer sets are listed in Table 1. For the RT-LAMP condition setup purpose, the LAMP amplification reaction at 65°C was monitored using the real-time machine FAM channel (CFX-96/ Bio-Rad, Harcules, CA), detecting fluorescence of GelGreen reagent. One cycle of the amplification was set as 1 minute; thus, the reaction time speed (min) was estimated to be equal to the Ct value. Melting curve analysis was performed following each LAMP reaction, and melting temperature (*T*_m_) was obtained to confirm the target-specific amplification.

### Realtime qRT-PCR detecting SARS-CoV-2 and influenza viruses as the reference tests

For the detection of the SARS-CoV-2 to compare with the RT-LAMP result, ‘N set no. 2 (N2)’ primers and TaqMan MGB probes designed by the National Institute of Infectious Diseases, Japan (NIID) were used as described before [16] with a slight modification of the reaction scale. The one-step real-time quantitative RT-PCR reaction mix consisted of 10.0 μl of QuantiTect Probe RT-PCR Master Mix (Qiagen), 1.0 μl of 10μM forward primer (final 500 nM), 1.4 μl of 10 μM reverse primer (700 nM), 0.8 μl of 10 μM TaqMan Probe (200 nM), 1.6 μl RNase-free water, and 2 μl of the RNA template. The RT step was performed at 50°C for 30 min, followed by incubation at 95°C for 15 min. The PCR step included 45 cycles of denaturation at 95°C for 15 sec, followed by annealing and extension at 60°C for 30 sec. The real-time amplification data were acquired and analyzed using ABI 7500 Fast real time PCR platform (Thermo Fisher Scientific). For the detection of influenza A and influenza B, 500 nM InfA-Fwd:5’-AAGACCAATCCTGTCACCTCTGA-3’, 500 nM InfA-Rev: 5’-CAAAGCGTCTACGCTGCAGTCC-3’, 250 nM InfA-Probe: 5’-FAM-TTTGTGTTCACGCTCACCGTGCC-BHQ-3’ and 500 nM InfB-Fwd: 5’-GAGACACAATTGCCTACCTGCTT-3’, 500 nM InfB-Rev: 5’-TTCTTTCCCACCGAACCAAC-3’, 250 nM InfB-Probe: 5’-FAM-AGAAGATGGAGAAGGCAAAGCAGAACTAGC-BHQ-3’ were used [17]. The amplification condition was the same as above.

### Multiplex reverse transcription-LAMP assay and the visualization of LAMP product by dipstick DNA chromatography

The multiplex RT-LAMP was performed in the same condition with a single RT-LAMP reaction as described above, except that three sets of primers listed in Table1 were all mixed, and primer concentration was optimized as described later. The DNA chromatography (C-PAS), blue-colored latex bead with streptavidin, and 300 mM NaCl C-PAS buffer ver2 were purchased from Tohoku bioarray (TBA). On the C-PAS strip, four tags cF1-4 were immobilized, which are the complementary sequences to the F1-F4 tag sequences of the primer. In this study, IAV, IBV, and SARS-CoV-2 LAMP amplified products are designed to bind to c-F1, c-F3, and c-F4 lines, respectively. One microliter of multiplex LAMP product, 17 μl of the C-PAS buffer ver2, and 2 μl of the latex beads were mixed (final 300 mM NaCl) in a 1.5ml tube or 96 deep-well plate, the DNA chromatography was inserted into the tube, and the result was confirmed after 10 minutes incubation by the naked eye. The condition of the C-PAS buffer was applied from the previously reported study [18].

### Making dried format multiplex reverse transcription-LAMP assay

The drying procedure of LAMP reagents was performed by our previously reported in-house protocol using trehalose [15] with slight modification for the concentration of RNase inhibitor for better prevention of the RNA degradation. The dried format multiplex RT-LAMP reagent consisted of two parts: enzymes and dNTPs on the tube lid and primers and detection dye on the bottom. This is because mixing all the reagents failed to keep enzyme activity in our attempt [15], possibly explained as making an amorphous phase for the enzyme vitrification is highly dependent on the successful dehydration [19, 20]. Three sets of primers listed in Table 1 (each 0.1 μl of 100 mM FIP with tag sequence and biotinylated BIP; 0.1 μl FIP and BIP without modification, 0.025 μl of 100 mM F3 and B3, 0.1 μl LF and LB), 1 μl of 3 mM CFI are dried with 0.7 μl of 2 M trehalose are prepared on the bottom of the 0.2 ml PCR tube. For the tube lid, 1.4 μl of 25 mM dNTPs, 0.05 μl of a high-concentration Bst-polymerase (120 U/μl; NEB), 0.25 μl of a normal concentration Bst-polymerase (8 U/μl), 0.3 μl of RNase inhibitor (40 U/μl; Takara Bio Inc., Shiga, Japan), 0.25 μl of WarmStart RTx reverse transcriptase (15 U/μl; NEB), and 1.6 μl of 2 M Trehalose was mixed, and placed inside of the tube lid. For aliquoting these reagents to the tube bottom and tube lid, an automatic pipette (E1-ClipTip; Thermo Fisher Scientific) was used to minimize liquid handling errors. The tube was air-dried with an ultra-low dew point air dryer (QD20-50; IAC Co., Kawasaki, Japan) for 12 hours. The dried LAMP reagent tube is kept with silica gel at ambient temperature for one week. Reaction buffer 23 μl, consisting of 20 mM Tris-HCl (pH10.0), 50 mM KCl, 6 mM MgSO4, 10 mM (NH_4_)_2_SO_4_ in TritonX-100, and 2 μl of template was added before the reaction.

## Results

### The sensitivity of newly designed RT-LAMP assays detecting SARS-CoV-2 RNA

The primer set for detecting SARS-CoV-2 was selected from ten candidate primer sets using synthetic DNA as a template, which showed the fastest amplification without non-specific amplification in the no-template control (NTC) (S1 Table and Table 1). Additionally, emerging nucleotide mutations in the SARS-CoV-2 genome that may affect the binding efficacy of LAMP primer sets were determined using the online COVID-19 CG tool [21] (https://covidcg.org/). Data from 11,207,833 sequences deposited in the GISAID from Dec 2019 to Jan 2023 were used. The confirmed nucleotide mutation in the primer region was relatively low, at highest 0.025%, suggesting that the designed LAMP primer sets using the original outbreak strain is still applicable to the current variant strains (S2 Table).

**Fig 1:**
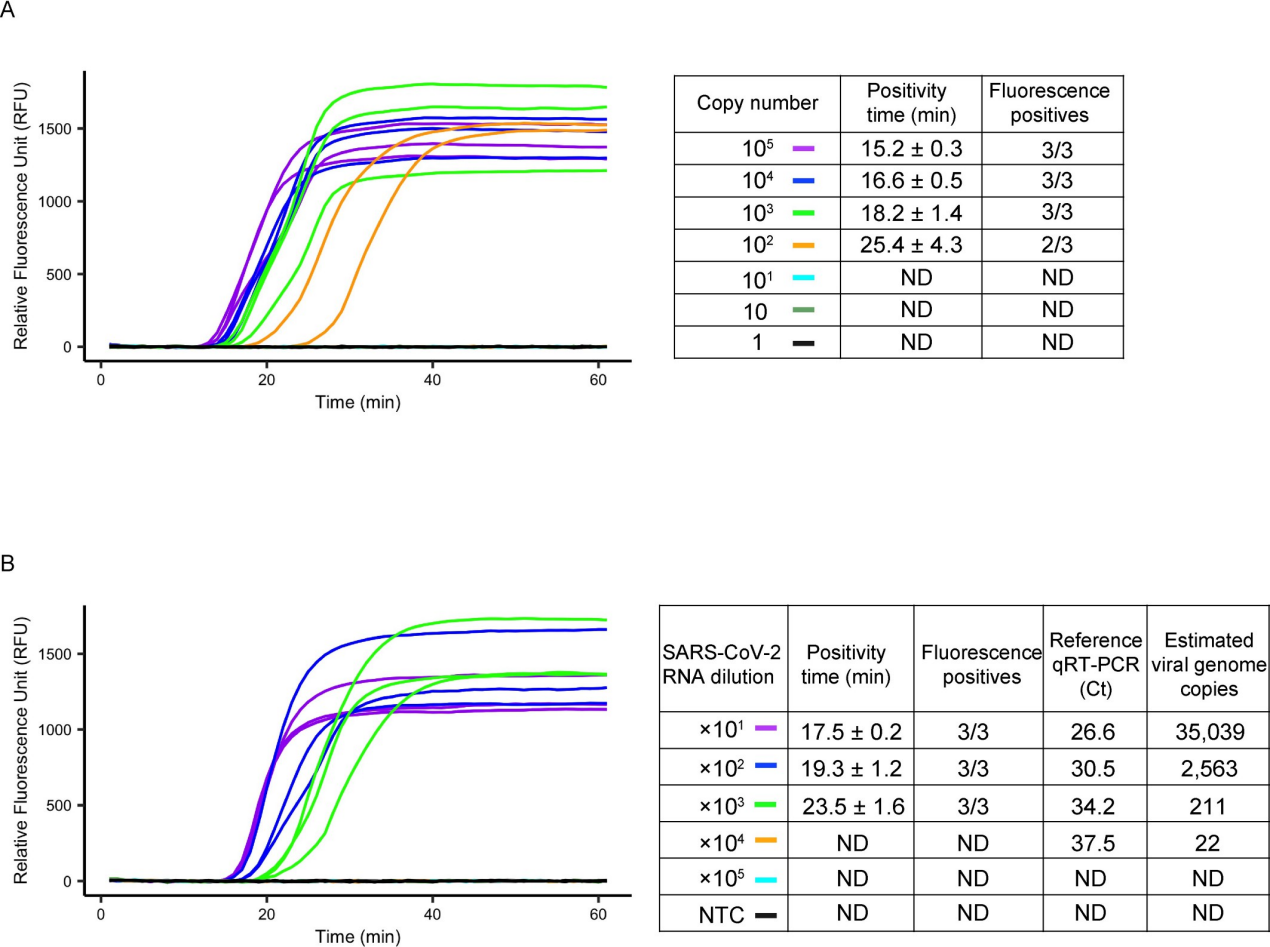
Evaluation of the sensitivity of the newly designed RT-LAMP assay in detecting SARS-CoV-2. (A) Amplification curves for the novel RT-LAMP assay using serially diluted plasmid DNA as a template. A conventional liquid format with a single primer set for detecting SARS-CoV-2 was used in the assay. Three independent experiments were performed. The mean ± SD value of the reaction Ct is shown as the positivity time (min). ND: not detected. (B) Amplification curves of the RT-LAMP assay using serially diluted viral RNA as a template. RNA extracted from the strain JPN/TY/WK-521 (Pango lineage A) was used. The same RNA was assayed for the reference qRT-PCR, and the mean cycle threshold (Ct) value of real-time RT-PCR assay and estimated copy number of the viral genome are shown.

Using the selected primer candidate, up to 1000 copies of DNA templates were amplified in 100% sensitivity (3/3; 95% CI: 0.29–1.00) for an amplification time of 18.2 ± 1.4 min (Fig 1A). 100 copies were also amplified in 66.6% (2/3; 95% CI: 0.09–0.99) for an amplification time of 25.4 ± 4.3 min. To further evaluate the analytical sensitivity, serially diluted RNA extracted from SARS-CoV-2 cultured viruses was assayed for RT-LAMP analysis. The same RNA was used for the real-time qRT-PCR assay (NIID-N2) as a reference test. The fluorescence signal generated from LAMP amplification was observed for an amplification time of 23.5 ± 1.6 min, detecting up to a 1000 times dilution point of the RNA (3/3; 95%CI: 0.29–1.00). This corresponds to the cycle Ct value of 34.2 in the reference real-time qRT-PCR, which was estimated to be 211 copies per reaction using the standard *in vitro* transcribed RNA as the standard (Fig 1B).

### Optimization of multiplex RT-LAMP condition detecting for SARS-CoV-2, influenza A, and influenza B viruses, and visualization on DNA chromatography

**Fig 2:**
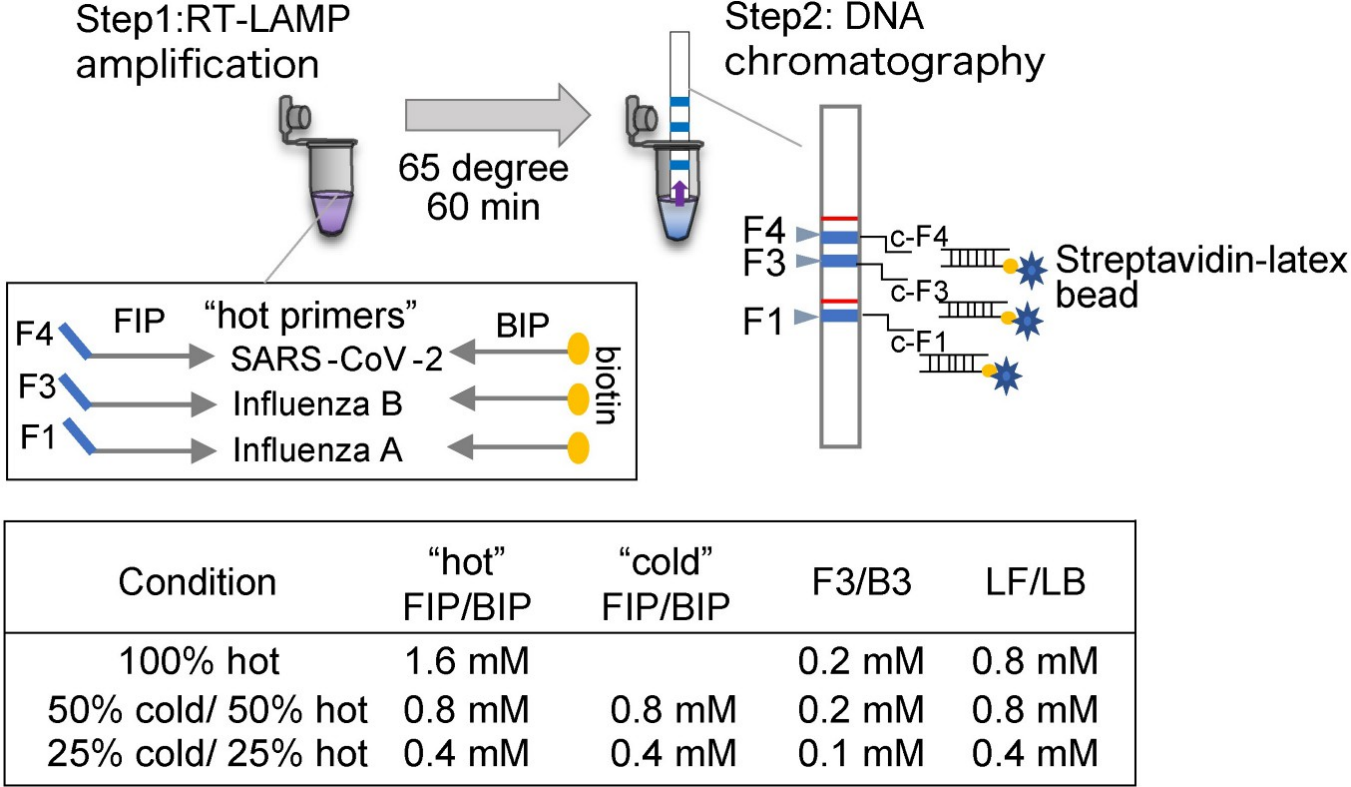
Schematic illustration of multiplex RT-LAMP and visualization using dipstick DNA chromatography. Step 1: Primers targeting SARS-CoV-2, IAV, and IBV are tagged with sequence Tags and biotin (“hot”) primers are mixed with non-modified primers (“cold”) primers in three different conditions. Step 2: After LAMP amplification, the product is visualized using DNA chromatography. C-F1, c-F3, and c-F4: complementary oligonucleotides for each tag. The blue lines represent the positive reactions, whereas the two red lines are the originally printed position markers.

To achieve multiplex RT-LAMP amplification and visual detection of SARS-CoV-2, IAV, and IBV; DNA chromatography C-PAS was utilized. The FIP primer for each target had a unique sequence oligonucleotide tag, and BIP had a biotin modification at the 5’-end. This enabled hybridization to the DNA chromatography, in which complement oligonucleotides were immobilized, and the streptavidin buffer generated a clear visible blue band on the strip (Fig 2).

**Fig 3:**
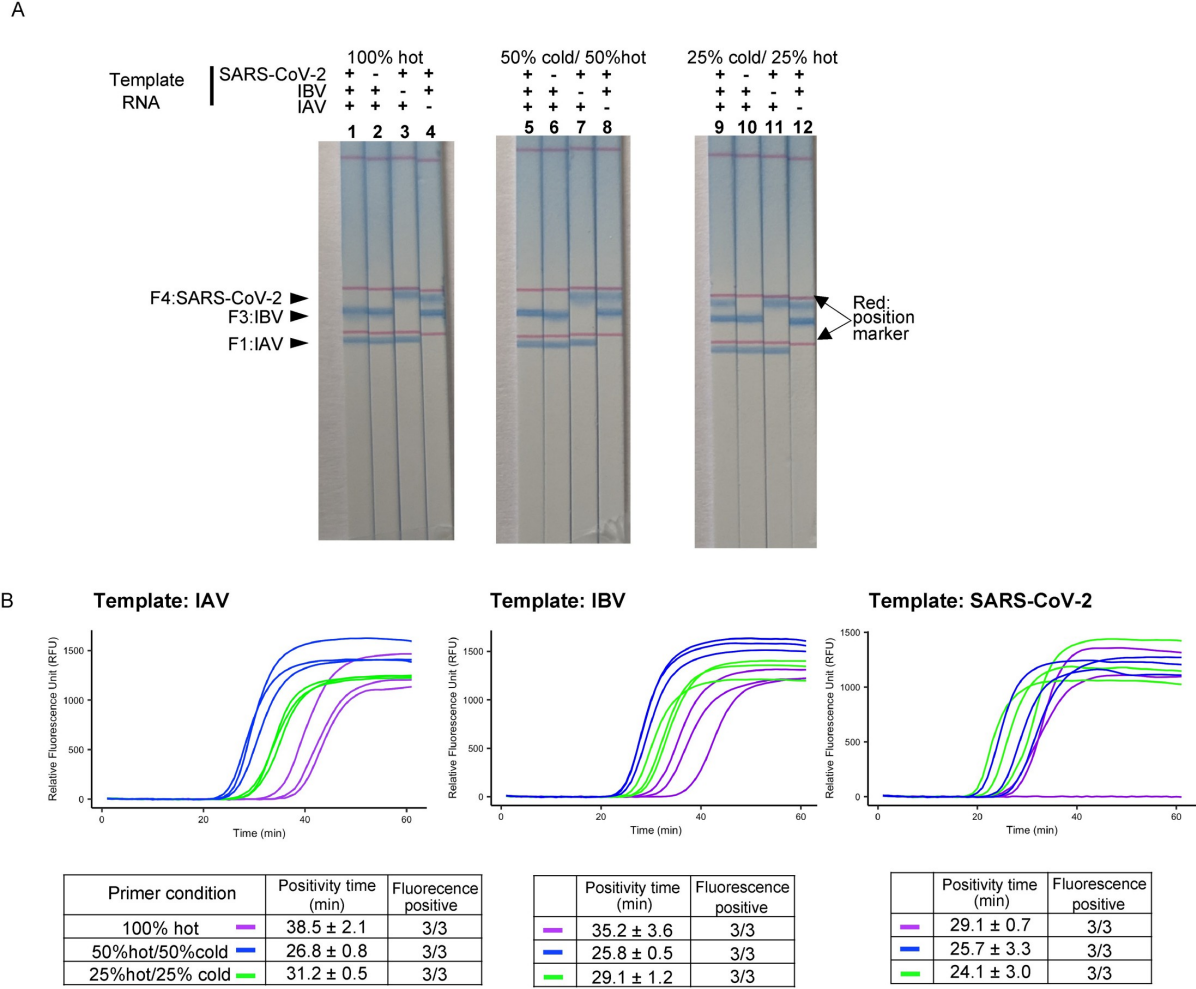
Optimization of the primer condition for multiplex RT-LAMP assay detecting SARS-CoV-2, IAV, and IBV. (A) DNA chromatography results visualizing multiplex RT-LAMP products. 1000 copies of SARS-CoV2, IAV, and IBV RNA templates were mixed, and used for testing the simultaneous amplification capacity. Three different primer conditions were compared by mixing “hot” (with tag and biotin) and “cold” (plain) primers. Blue bands are the positive reactions. Arrowhead indicates the positive position for each LAMP product. The red lines are the position marker. The experiment was performed three times, and a representative of the same result is shown. (B) The amplification curve and the positivity time of the RT-LAMP in three different primer conditions detecting a single RNA template. The mean ± SD value of the reaction Ct is shown as the positivity time (min).

Next, we mixed three sets of primers detecting SARS-CoV-2, IAV, and IBV (a total of 20 primers are shown in Table 1) to make a multiplex RT-LAMP system. In this optimization experiment, the synthesized RNAs were used as templates at a concentration of 1000 copies per reaction. Amplification was monitored by fluorescence using a real-time PCR machine, and visualized using DNA chromatography after 60 min of amplification. First, the primer amount of the single LAMP amplification for three targets was all mixed, in which FIPs were tag sequence-labeled, and BIPs were biotin-labeled (100% hot primer condition; primer concentration are shown in Fig 2). Following multiplex LAMP reaction and DNA chromatography visualization, a colored band was observed for the two target templates (Fig 3A; 2–4). However, a line corresponding to SARS-CoV-2 amplification was not observed when the three targets were used as templates (Fig 3A: 1). This negative result on DNA chromatography in the simultaneous detection of the three targets was possibly due to poor SARS-CoV-2 amplification under multiplexing conditions, or poor amplicon-chromatography hybridization due to interference of multiple amplicons. The latter is more likely as the amplified products from three single targets showed a positive result for each target and resulted in negativity for the SARS-CoV-2 position when mixed and visualized using DNA chromatography. Thus, the condition of the half amounts of primers for modified (“hot”) primers and the remaining half for without tag-sequence and biotin (“cold”) primers was tried (50% hot/ 50% cold), resulting in a similar result (Fig 3A; 5–8). However, a faster amplification speed was observed when compared with the primer condition without cold primers (Fig 3B; 100% hot vs. 50% cold/ 50% hot); thus, we hypothesized that tag sequences with spacer C3 in FIP primers may limit the amplification efficacy of the RT-LAMP reaction. Then, the 25% cold/ 25% hot condition was tested, in which FIP/BIP primers without tag-sequence and biotin (cold primer) were mixed with the hot primers at a concentration ratio of 1:1 while the primer amount for each target was kept as half. This primer condition provided a balanced result for detecting all three targets on the chromatography (Fig 3A; 9–12) and was chosen as the primer condition for our multiplex RT-LAMP system.

### The analytical sensitivity and specificity of the air-dried multiplex RT-LAMP in detecting SARS-CoV-2, IAV, and IBV simultaneously

**Fig 4:**
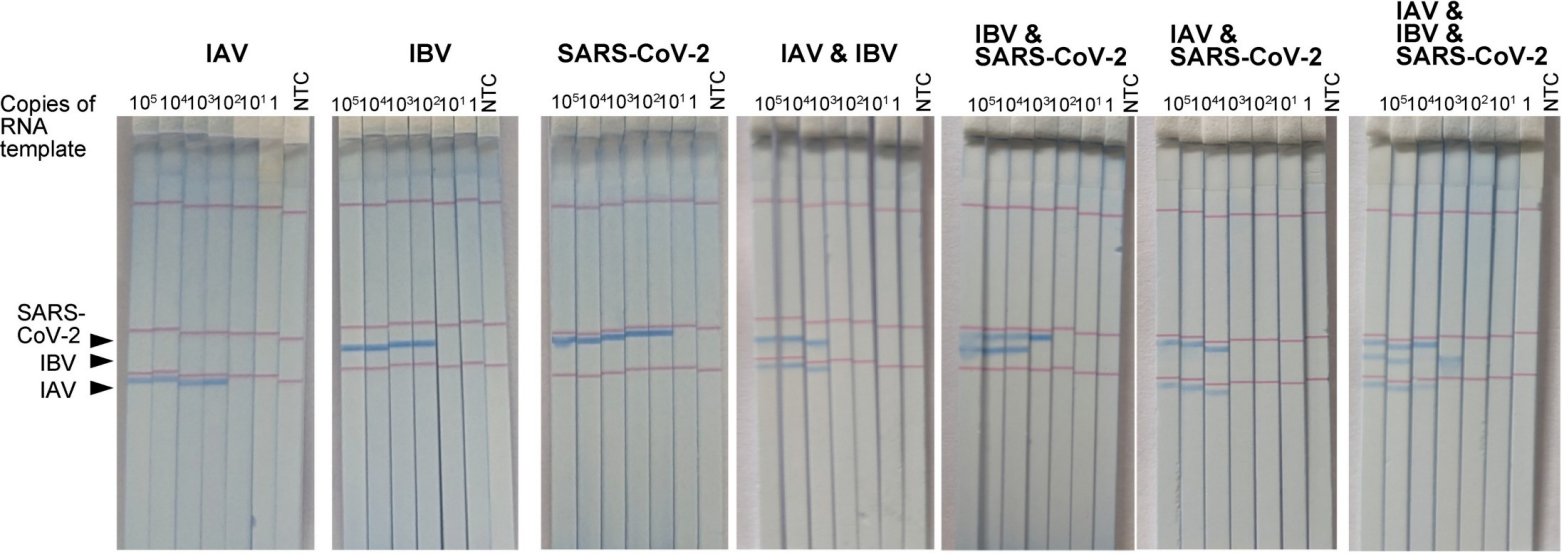
Analytical sensitivity of dried multiplex-LAMP and DNA chromatography detecting SARS-CoV-2, IAV, and IBV. Representative result of DNA chromatography visualizing dried multiplex-LAMP products from three independent experiments. A single or combination of SARS-CoV2, IAV, and IBV RNA was used as the multiplex RT-LAMP assay template. The remaining results with amplification times are summarized in Table 2. The blue bands represent a positive reaction and the arrowhead indicates a positive position for each LAMP product. The red line is the position marker.

Under liquid-format RT-LAMP conditions, the reagent preparation procedure is laborious, which contains 26 primers and 9 reagents that require strict cold temperature storage monitoring. Thus, to minimize the procedure and for the convenience of the transport and storage, we applied our previously developed air-drying technique to the multiplex RT-LAMP system using trehalose as a preserving molecule. A mixture of primers and reagents was air-dried at room temperature in a PCR tube. The dried tubes were tested after a week to validate the analytical sensitivity of the multiplex RT-LAMP assay. To test the analytical sensitivity of the multiplex RT-LAMP assay for detecting three targets, SARS-CoV-2, IAV, and IBV, 10-fold serially diluted RNA templates containing 10^5^–1 copies per reaction were prepared and assayed using the multiplex RT-LAMP assay under the primer conditions described above. At a dilution of 100 copies, three out of three replicates of IBV and SARS-CoV-2 were positive both in fluorescence and C-PAS (100% sensitivity; 95% CI: 0.29–1.00). while two out of two were positive for IAV (66.6% sensitivity; 95% CI: 0.09–0.99: Fig 4 and Table 2). The observed sensitivity of 100 copies of RNA was similar to that obtained by real-time PCR estimation of SARS-CoV-2 viral RNA (up to 211 copies in Fig 1B). The sensitivity of the single RT-LAMP assay for IAV and IBV also agreed with the analytical sensitivity of 25–250 copies/reaction reported for the same primer sets [13]. However, when multiple targets were amplified together in the multiple RT-LAMP system, a 10–100 fold less sensitivity was observed (Fig 4 and Table 2), which may be explained by the competition of the enzymes and interference of the reactions.

**Table 2:** The sensitivity of the multiplex LAMP system using the DNA chromatography, and the positivity time in fluorescence.

		Template RNA copies						
RNA templates	Chromatography results/Amplification time	10^5^	10^4^	10^3^	10^2^	10^1^	1	NTC
Single Target	IAV	3/3	3/3	3/3	2/3	0/3	0/3	0/3
	Time (min)	25.9 ± 3.2	28.2 ± 1.4	33.4 ± 4.9	37.4 ± 11.5	ND	ND	ND
	IBV	3/3	3/3	3/3	3/3	0/3	0/3	0/3
	Time (min)	23.2 ± 0.7	25.7 ± 1.2	30.5 ± 1.9	38.6 ± 6.0	ND	ND	ND
	SARS-COV-2	3/3	3/3	3/3	3/3	0/3	0/3	0/3
	Time (min)	14.5 ± 0.8	16.4 ± 2.0	18.0 ± 2.0	21.5 ± 1.9	ND	ND	ND
Two Target	IBV	3/3	3/3	3/3	0/3	0/3	0/3	0/3
	IAV	3/3	3/3	3/3	0/3	0/3	0/3	0/3
	Time (min)	23.8 ± 0.5	31.4 ± 3.3	33.1 ± 0.8	ND	ND	ND	ND
	SARS-CoV-2	3/3	3/3	3/3	3/3	0/3	0/3	0/3
	IAV	3/3	3/3	3/3	3/3	0/3	0/3	0/3
	Time (min)	16.6 ± 0.2	19.0 ± 0.6	21.5 ± 0.7	40.6 ± 3.6	ND	ND	ND
	CoV-2	3/3	3/3	3/3	2/3	0/3	0/3	0/3
	IBV	3/3	3/3	3/3	1/3	0/3	0/3	0/3
	Time (min)	16.5 ± 0.6	19.4 ± 0.5	23.4 ± 1.9	29.5 ± 2.1	ND	ND	ND
Three Target	SARS-CoV-2	3/3	3/3	3/3	0/3	0/3	0/3	0/3
	IBV	3/3	3/3	0/3	2/3	0/3	0/3	0/3
	IAV	3/3	3/3	1/3	1/3	0/3	0/3	0/3
	Time (min)	16.6 ± 0.2	19.0 ± 0.6	21.5 ± 0.7	40.6 ± 3.6	ND	ND	ND

The positivity for each line in DNA chromatography was determined after 60 min of RT-LAMP amplification and is shown as a positive number out of three. The fluorescence positivity time was determined as the Ct value of the LAMP amplification, in which one cycle was set to one minute. All experiments were performed in triplicates, and the mean ± SD value of the positivity time is shown.

In all NTC experiments (n = 21, Table 2) within an amplification time 60 min, none of them showed a non-specific reaction in fluorescence and DNA chromatography, thus the determined specificity of the multiple RT-LAMP system under this condition was 100% (0/21; 95% CI: 0–0.16). The specificity of the positive reactions was also confirmed by the *T*_m_ values after amplification, where positive samples from IAV, IBV, and SARS-CoV-2 showed *T*_m_ values of 87.5, 86.5, and 86.5, respectively (S1A Fig). When the reaction time was extended to 150 min, three out of 24 reactions were positive for NTC, which was assumed to be non-specific amplification or primer dimer formation. Non-specific amplifications were observed after 93.5 minutes, with sufficient time remaining from the decided LAMP reaction endpoint of 60 min (S1B Fig). The chromatography results of these three non-specific amplifications were inconsistent; one showed a double band on the IAV, SARS-CoV-2 line, and the other two showed no band on chromatography (S1C Fig), which could be explained by the different combinations of primer-dimers in each non-specific amplification.

#### Evaluation of multiplex RT-LAMP using pseudo-clinical samples pulsed with influenza virus

To determine the sensitivity and specificity of IAV and IBV detection, pseudo-clinical specimens were prepared by mixing known titers of IAV and IBV into nasopharyngeal swab samples. IAV and IBV virus supernatants were added to the swab sample to obtain the highest concentration of 1.43 × 10^5^ PFU/ml, which is equivalent to 1000 PFU in 2 μl of template RNA, assuming the RNA extraction recovery rate was 100%. A ten-fold dilution of the virus was prepared, and the RNA extracted from these pseudo-clinical influenza samples was used for the dried format multiplex RT-LAMP system. qRT-PCR was also performed as a reference test. As a result, in both IAV and IBV, 100 PFU equivalent samples were amplified in three out of three independent experiments (Table 3, 100% sensitivity; 95% CI: 0.29–1.00). In IBV, 10 PFU equivalent samples were also amplified in two out of three experiments (Table 3, 66.6% sensitivity; 95% CI: 0.09–0.99).

**Table 3:** Result of dried format multiplex LAMP using RNA extracted from nasopharyngeal samples pulsed with influenza virus.

Virus strain	Virus equivalent in LAMP reaction	Chromatography positives	Time (min)	Reference qRT-PCR Ct value
A/Hokkaido/32/H1	1000 PFU	3/3	29.5 ± 0.7	23.1
	100 PFU	3/3	30 ± 3.5	26.9
	10 PFU	0/3	ND	31.9
	1 PFU	0/3	ND	34.1
	0.1 PFU	0/3	ND	36.8
	0.01 PFU	0/3	ND	39.8
	0.001 PFU	0/3	ND	ND
	NTC	0/3	ND	ND
B/Hokkaido/2/2016	1000 PFU	3/3	30.7 ± 2.1	24.5
	100 PFU	3/3	34.1 ± 1.0	27.7
	10 PFU	2/3	37.4 ± 3.8	30.3
	1 PFU	0/3	ND	33.9
	0.1 PFU	0/3	ND	37.3
	0.01 PFU	0/3	ND	ND
	0.001 PFU	0/3	ND	ND
	NTC	0/3	ND	ND

The positivity for each line in DNA chromatography was determined after 60 min of RT-LAMP amplification and is shown as a positive number out of three. The mean ± SD value of fluorescence positivity time is shown. Reference qRT-PCR assay was performed using the same RNA, and the obtained Ct value is shown.

### Evaluation of multiplex RT-LAMP using COVID-19 clinical specimens

**Fig 5:**
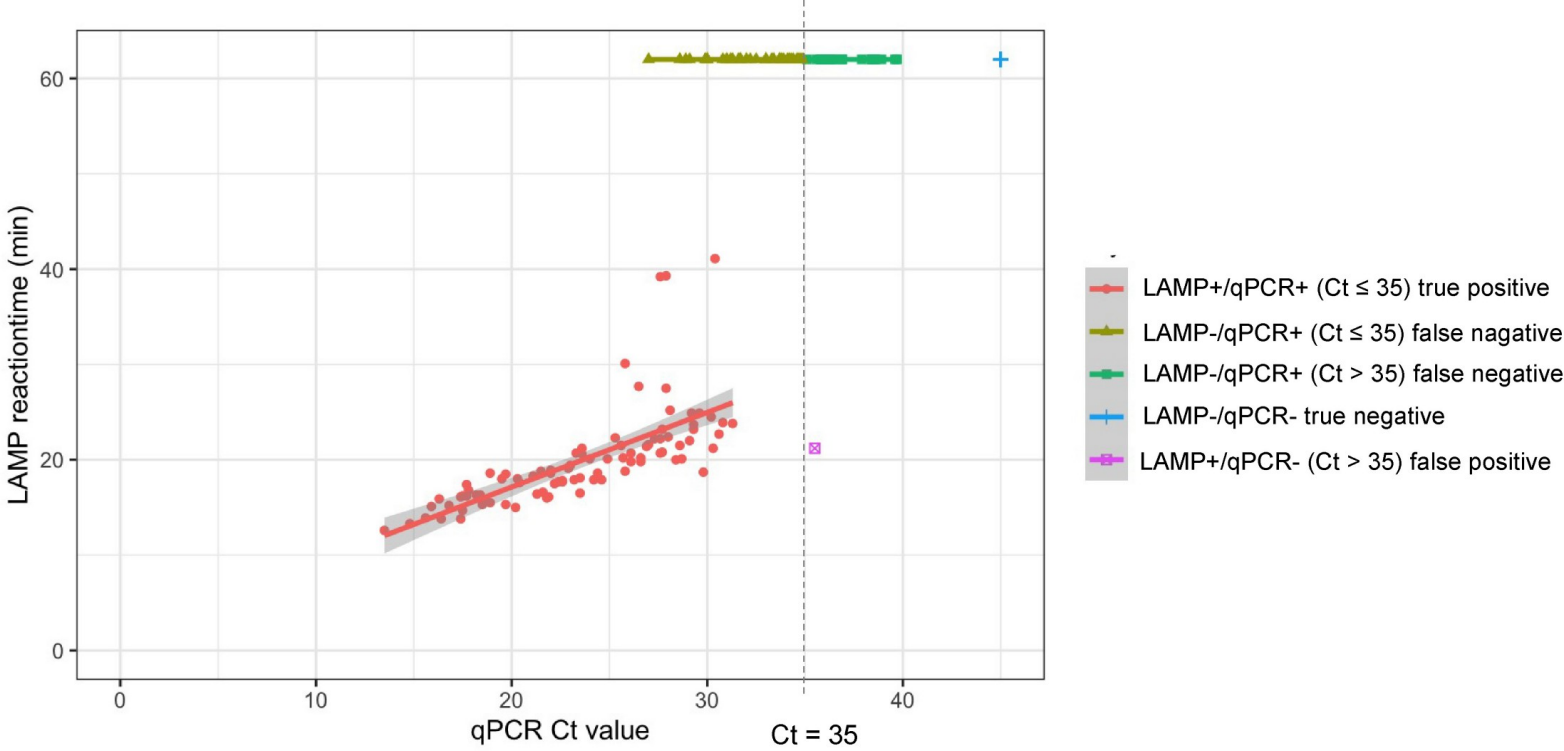
Reactivity of multiplex LAMP compared with reference qRT-PCR for detecting clinical COVID-19 suspected specimens. (A) The scatter plot of the reaction time of dried format multiplex LAMP using clinical COVID-19 suspected specimens plotted against the Ct value of NIID-N2 reference qRT-PCR. The results of qPCR and LAMP were categorized and shown in different colors. Ct threshold values of 35 and 40 were used for sensitivity/specificity determination. A linear regression line with a shaded SE value is shown for the result group LAMP+/qPCR+ (Ct ≤ 35). (B) DNA chromatography results after multiplex LAMP assay.

The newly developed multiplex RT-LAMP system was evaluated using 192 RNA samples from nasopharyngeal swabs collected from patients with suspected COVID-19 in Ireland. RNA was tested using real-time qRT-PCR for SARS-CoV-2, IAV, and IBV as a reference test, and the same RNA was used to validate the multiplex RT-LAMP system in a dried format. All 192 samples tested negative for the influenza virus. The LAMP reaction was monitored using a real-time PCR machine for 60 min, and the positive turnaround time was plotted on the Y-axis, whereas the positive real-time qRT-PCR results were plotted as Ct values on the X-axis (Fig 5). All the multiplex RT-PCR amplified products were also tested for DNA chromatography C-PAS, and the positive results for COVID-19 agreed with the LAMP positivity results measured by fluorescence (S2 Fig). In the multiplex LAMP assay, a positive correlation was observed between the RT-LAMP reaction time and qRT-PCR Ct value, suggesting that the RT-LAMP reaction time is correlated with the original viral RNA amount, although LAMP amplification is not a strictly proportional event. A false-negative result in RT-LAMP was observed for the sample above Ct 27 (Fig 5), suggesting a lower detection power for samples with low viral RNA.

The determined sensitivity for dried format multiplex RT-LAMP assay using the clinical COVID-19 specimen was 61% (95% CI: 0.53–0.69), and specificity was 100% (95% CI: 0.92–1.00) when all positive results under a Ct value of 40 in qRT-PCR were considered as positive. Alternatively, when the Ct values of 35 were used as a cut-off value, the sensitivity and sensitivity were determined to be 71% (95% CI: 0.62–0.79) and 99% (95% CI: 0.92–1.00), indicating that moderate sensitivity and high specificity can be obtained for the higher viral RNA road specimen (Table 4).

**Table 4:** Result of dried format multiplex LAMP using clinical COVID-19 suspected specimens, and its agreement with the reference qPCR test.

	Threshold Ct value for qPCR: Ct< = 40			Threshold Ct value for qPCR: Ct< = 35		
	qPCR positive	qPCR negative		qPCR positive	qPCR negative	
LAMP positive	90	0	Sensitivity: 61% (95%CI: 0.53–0.69)	89	1	Sensitivity: 71% (95%CI: 0.62–0.79)
			Positive predictive value: 100% (95% CI: 0.96–1.00)			Positive predictive value: 99% (95% CI: 0.94–1.00)
LAMP negative	57	45	Specificity: 100% (95% CI: 0.92–1.00)	36	66	Specificity: 99% (95% CI: 0.92–1.00)
			Negative predictive value: 44% (95% CI: 0.34–0.54)			Negative predictive value: 65% (95%CI: 0.55–0.74)

## Discussion

Both COVID-19 and influenza cause respiratory illnesses with similar clinical manifestations; therefore, a differential diagnosis is difficult without laboratory confirmation. During the pandemic, the cases of influenza virus infection have decreased. This is possibly a result of strict interventions undertaken by most countries, such as physical distancing, travel restrictions, and staying home [22, 23]. In addition, clinical diagnosis often relies on a single laboratory test for COVID-19. This may have decreased the detection rate of influenza infections. However, seasonal and pandemic influenza viruses can cause concurrent COVID-19 epidemics. Co-infection with SARS-CoV-2, IAV, and IBV has also been reported [23] and their potential to worsen the clinical outcome of COVID-19 has been suspected [24, 25]. Therapeutic drugs for COVID-19 have recently been developed (WHO-2019-nCoV-therapeutics-2022.4-eng.pdf) and are now available, highlighting the increasing importance of specific diagnoses and treatments. Thus, to detect these two important respiratory viruses simultaneously in a field-deployable format, a multiplex LAMP assay that can detect SARS-CoV-2, IAV, and IVB was developed in this study.

In addition to the gold-standard real-time qRT-PCR and RDTs, many new methods for COVID-19 diagnosis have been developed. LAMP is one such method, and commercial products are available from several companies. However, these are mostly single-pathogen detecting methods. A recent study described a multiplex LAMP method or qRT-PCR to detect SARS-CoV-2 and influenza viruses simultaneously. This is achieved using multicolor fluorescence and/or differentiation using melting curve analysis. This technology still requires an initial cost for the fluorometer [6]. The advantage of our multiplex LAMP system is that the required equipment is minimal, making it suitable for use in resource-limited areas. In addition, the biggest challenge in the use of conventional LAMP is the requirement for a cold chain. To solve this problem, we utilized our in-house protocol for drying reagents with trehalose, which is ideal for LAMP in clinical settings [26]. We have previously shown that enzyme stability in this method can be guaranteed for up to six months, with a slight decrease in reaction time. The storage at 10°C prolonged its shelf-life [27].

Our results using 192 clinical specimens indicate that our developed multiplex RT-PCR has lower sensitivity and faster diagnostic performance for detecting SARS-CoV-2 than conventional RT-qPCR. Notably, a clear correlation was observed between the Ct value of RT-qPCR and the reaction time of our RT-LAMP assay, especially in the high viral-load samples with a lower Ct values. In the RT-LAMP assay, samples with Ct values < 27 were all detected. The Ct value alone cannot be compared to the viable viral load, but it has been argued that the culture positivity declines with increasing cycle threshold values, and above 35 cycles may not be contagious [28]. Although more data are required to determine the real threshold for each qRT-PCR test, it is important to consider the test accuracy by comparing the Ct values. Therefore, in this study, we calculated the sensitivity and specificity of qRT-LAMP test for each Ct threshold. In either criterion, our multiplex qRT-LAMP test showed moderate sensitivity with 61% (95% CI: 0.53–0.69) for the Ct<40 threshold and 71% (95% CI: 0.62–0.79) for the Ct<35 threshold. Importantly, the specificity was excellently high, except for one sample that was categorized as a false positive in LAMP when a qRT-PCR Ct<35 threshold was applied. This result suggests that our qRT-LAMP assay is useful for rapid and reliable diagnosis, particularly in primary healthcare settings.

This study has several limitations. First, we validated only purified RNA samples from suspected COVID patients. To be truly point-of-care testing, RNA extraction step should be omitted. In related research, several efforts have been made to improve the sensitivity and specificity of unprocessed samples. The prevention of endogenous RNase activity appears to be a key steps in preventing unreliable results. Boiling of the samples with reducing agents such as tris(2-carboxyethyl)phosphine (TCEP) to inactivate endogenous RNase, and simple glass milk purification from nasopharyngeal swab samples has been reported to show dramatically improved sensitivity and specificity [29]. Such a trial using crude sample is required for point-of-care testing in resource-limited places. In addition, as we only used COVID-19-positive specimens, a test of the clinical samples that are positive for influenza viruses, and/or coinfected with SARS-CoV-2 and influenza viruses is also required in the future. Another concern is the relatively low target detection sensitivity. The analytical sensitivity of the synthesized RNA was 100 copies for the IAV/IBV/SARS-CoV-2 single target, whereas it was 10 PFU and 100 PFU for the spiked IBV and IAV pseudo-specimens, respectively. This was obviously less sensitive than qRT-PCR methods [30, 31]; thus, further improvement in the primer design of the assay system is needed. The concern regarding the operational procedure to avoid the carry-over risk of amplicon contamination also needs to be addressed by creating a closed container system.

## Conclusions

A multiplex LAMP assay combined with DNA chromatography was developed for COVID-19 and influenza. The total time from RNA to the results was less than 60 min. The result is easily visible without any equipment. Therefore, our multiplex LAMP assay may be a useful, time-saving, and field-deployable diagnostic method for these diseases. The multiplex LAMP assay developed in the present study can be applicable for various disease combinations.

## Supporting information

S1 Table The primer screening results for detecting SARS-CoV-2 nucleocapside region. For an initial primer candidate screening, ten candidate primer sets were tested using synthesized DNA. The best performance primer set 1, which showed the fastest amplification without non-specific amplification was selected.
(XLSX)

S2 Table The observed mutation frequencies in the primer region. The mutation data of 11,207,833 sequences from Dec 2019 to Jan 2023 was obtained from COVID-19 CG tool.
(XLSX)

S1 Fig Specificity of the multiplex LAMP assay and DNA chromatography. (A) Melting curve analysis of the multiplex LAMP products after an extended reaction time of 150 min. The positive RNA control for IAV, IBV, and SARS-CoV-2 in 1000 copies, and n = 24 no template controls (NTCs) were tested. Three non-specific amplifications from the NTC showed different *T*_m_ values from the positive controls. (B) Amplification plots of n = 24 NTC samples. (C) Results of DNA chromatography in three non-specific amplifications.
(TIF)

S2 Fig DNA chromatography results after multiplex LAMP detecting clinical COVID-19 suspected specimens. The chromatography results for 192 of COVID-19 suspected specimens after multiplex LAMP.
(TIF)

S1 File Inclusivity in global research. (DOCX)
