# Supplementary figures and images for: Field-deployable multiplex detection method of SARS-CoV-2 and influenza virus using loop-mediated isothermal amplification and DNA chromatography
Source: PLoS One. 2023 May 16;18(5):e0285861. doi: 10.1371/journal.pone.0285861 (PMC10187927; doi:10.1371/journal.pone.0285861)

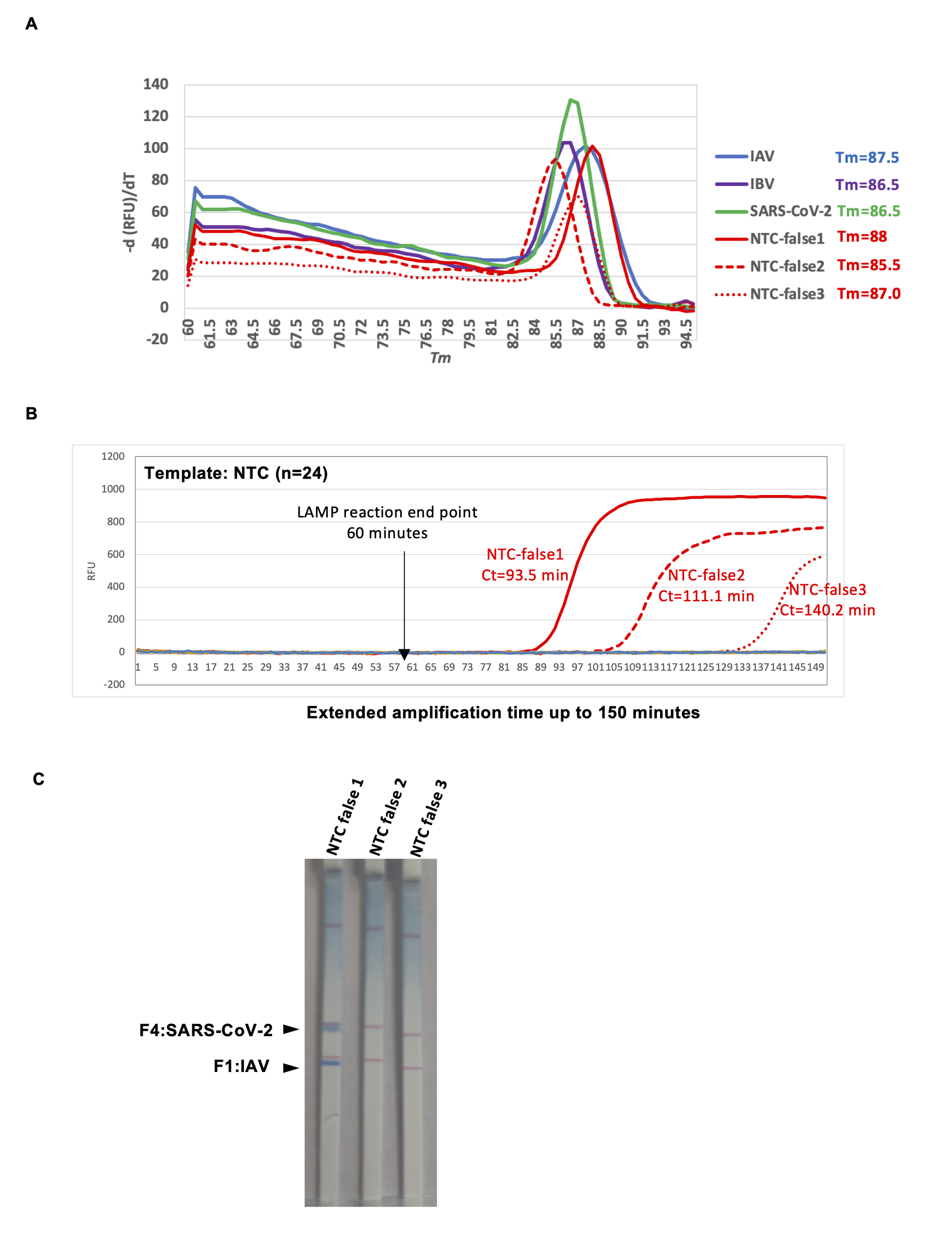

Supplement: S1 Fig — (A) Melting curve analysis of the multiplex LAMP products after an extended reaction time of 150 min. The positive RNA control for IAV, IBV, and SARS-CoV-2 in 1000 copies, and n = 24 no template controls (NTCs) were tested. Three non-specific amplifications from the NTC showed different Tm values from the positive controls. (B) Amplification plots of n = 24 NTC samples. (C) Results of DNA chromatography in three non-specific amplifications. (TIF) [file pone.0285861.s003.tif]

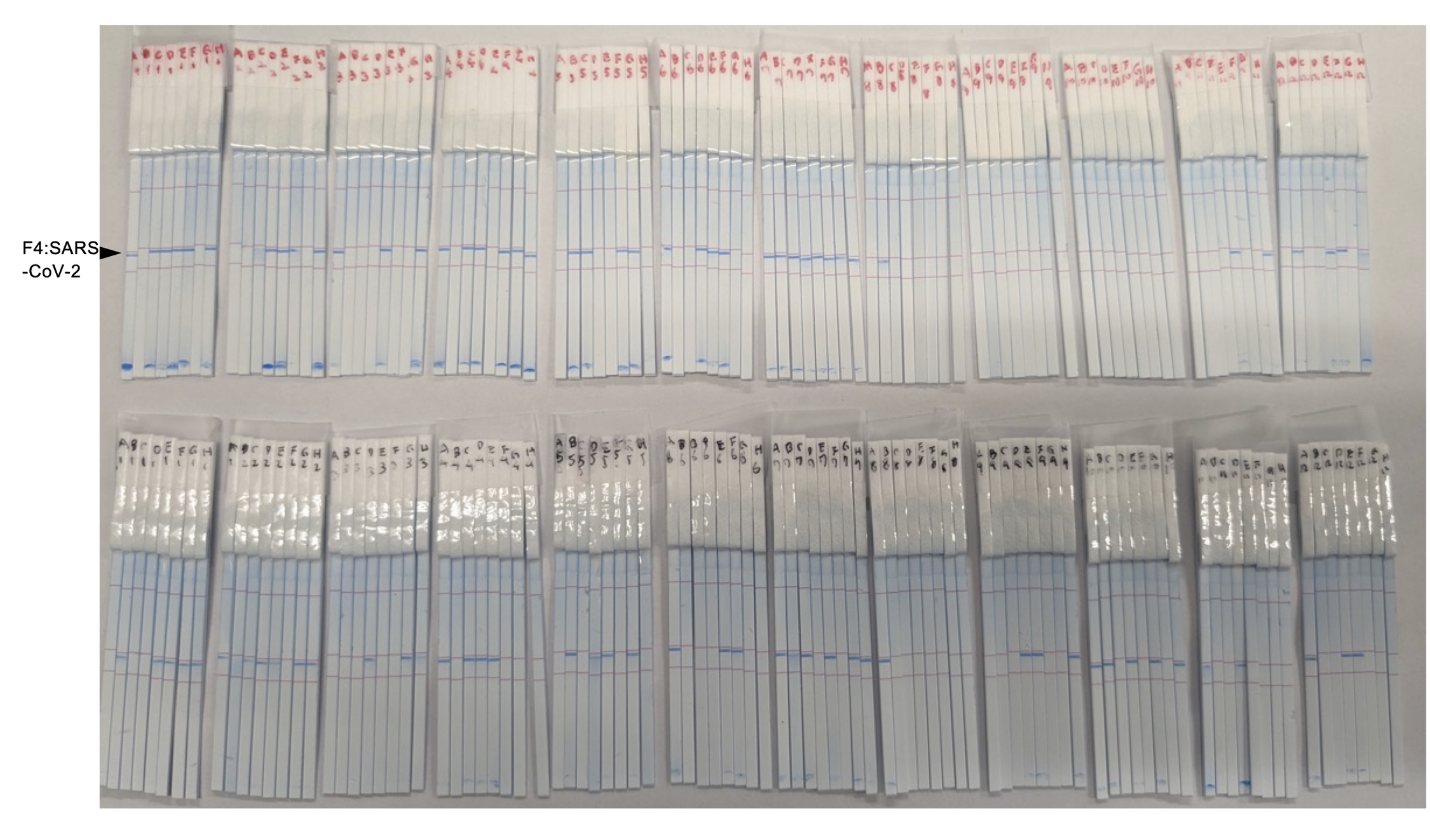

Supplement: S2 Fig — The chromatography results for 192 of COVID-19 suspected specimens after multiplex LAMP. (TIF) [file pone.0285861.s004.tif]
